# Emotional intelligence and the Occupational Personality Questionnaire (OPQ)

**DOI:** 10.3389/fpsyg.2014.00935

**Published:** 2014-09-15

**Authors:** Adrian Furnham, Mary-Clare Race, Adrienne Rosen

**Affiliations:** Department of Clinical, Educational, and Health Psychology, University College LondonLondon, UK

**Keywords:** Emotional Intelligence, self concept, other person perception, personality inventory, mood

## Abstract

This study explores the relationship between the Bar-on EQ-I and the Occupational Personality Questionnaire OPQ32i to determine if there is a link between self- and other-reported Emotional Intelligence and personality traits. Data was obtained from 329 managers working in the IT and Finance sectors and included multi-source (360°) measures of Emotional Intelligence. Results indicated construct overlap and correlations between some elements of Emotional Intelligence and the OPQ32i with a stronger relationship between 360 measures of Emotional Intelligence and personality. On both the self-report measure of EQ-I and the 360 measure the mood scale showed a strongest link with personality factors. Measures of Emotional Intelligence which include a 360 component may thus provide a more useful indicator of an individual's ability to manage their own feelings and those of others.

## Introduction

There is a rapidly growing literature on Emotional Intelligence: definition, measurement, origin, and consequences (Matthews et al., 2002; Murphy, 2006). The idea of emotional intelligence is discussed and debated widely within the mainstream business world with a growing claim that it can be a better predictor of high performance than the traditional measure of general intelligence (Furnham, 2008a; Weinberger, 2009).

Measures of emotional intelligence vary widely in both content and method of assessment and tend to fall into two broad camps; those that focus on *ability* and are derived from the original EI conceptualization (with a focus on being able to reason validly about emotions) and measures of *self-report* (trait) (which focus on the behavioral dispositions and self perceptions of one's ability to recognize and understand emotions (Petrides and Furnham, 2001, 2003). Empirical work in this field would suggest that trait EI is more robustly related to, but distinct from, personality as measured by the Big Five (Petrides and Furnham, 2001) and is quite different from ability EI (Petrides et al., 2007). There are, however, personality correlates of ability measures of EQ, particularly Agreeableness (Fiori and Antonakis, 2011).

Critics of ability measures of EI highlight the misconception that EI or competencies can be measured through self-report (Cooper and Petrides, 2010) and the potential for faking on self-report EI measures. Similar criticisms of the measure used in this study have been made (Petrides et al., 2007).

Other views position EI, and in particular trait EI, as an individual difference construct and therefore argue that EI exists within the same space as personality accounting for very little criterion variance above and beyond that of basic personality dimensions (Matthews et al., 2002). Petrides et al. (2007) explore this in more detail and, in their study found evidence to suggest that EI is a distinct construct, partially because it is determined by a number of personality factors and “exists at the lower levels of personality hierarchies” (p.48) thus somewhat dispelling the myth that EI is simply personality “rebranded.”

This study focuses on the relationship between two popular measures of emotional intelligence and personality in business settings though neither are used in mainstream psychological research on individual differences (Furnham, 2008a). Both are embedded in a separate theoretical framework but appear to have considerable conceptual overlap. The central question in this study is the empirical relationship between these two measures.

### The Bar-On measure

The measure used in this study is the Bar-On Emotional Quotient Inventory (EQ-i), which is designed to measure competencies including awareness, stress tolerance, problem solving, and happiness (Bar-On, 1997). Doubts have been expressed about the Bar-On model in the literature especially the factorial structure of the test and the selection of facets (Petrides et al., 2007). Matthews et al. (2002) noted that the theory behind the measure is vague and others have found the model to be highly susceptible to faking (Grubb and McDaniel, 2007; Day and Carroll, 2008). However, despite these criticisms the model continues to be applied in a variety of academic and real world settings (Lievens et al., 2011). Furthermore, the measure has been shown to have acceptable psychometric properties like internal consistency, convergent validity, and resistance to response style and bias (Dawda and Hart, 2000). Various attempts have been made to assess various different versions and translations of the measure with different degrees of support for such issues as factor structure (Kun et al., 2012) and concurrent validity (Al Said et al., 2013).

### The OPQ personality measure

Measurement of personality has equally attracted vast amounts of attention and many commercial organizations, rather than academic institutions, have developed measures which are now commonplace in the HR professional's toolkit. One such measure that has expanded greatly over the past 20 years all around the world is Saville et al. (1984) who claim several advantages for their “Occupational Personality Questionnaire” (OPQ) derived in part from the 16PF. The most comprehensive versions of the OPQ measure 30 scales which are grouped into three categories, associated with *Relationships with People*, *Thinking Style*, and *Feelings and Emotions*, respectively. Robertson and Kinder (1993) carried out a meta-analysis of some 21 different populations who had all completed the OPQ. They showed that, if specific hypotheses were tested, there was strong evidence of the criterion-related validity of the OPQ. It continues to attract attention with a recent study yielding a six factor solution interpretable within the Five Factor Model space (Woods and Hardy, 2012).

Early evaluations of the OPQ were critical (Barrett et al., 1996) however the measure has undergone significant development and an extenstive report by the British Psychological Society concluded that there was good evidence of the tests norms, reliability as well as construct and criterion-related validity (British Psychological Society, 2007).

### Personality and emotional intelligence

This study is concerned with the degree of association between a popular model of personality and the Bar-on model of emotional intelligence. In the current study we used two EQ measures: the original self-report measure and the 360 measure where different raters—typically a manager (boss), peers, direct reports, and others rate a specific person. It has been suggested that observer data is less prone to dissimilation and hence more accurate (Furnham, 2008a,b).

Van der Zee et al. (2002) present a summary of early findings which point to a stronger relationship between emotional intelligence and personality than the relationship between emotional intelligence and general intelligence. In a study to determine the relationship between emotional intelligence, cognitive ability, and personality with academic achievement Newsome et al. (2000) found that many of the factors in the Bar-on measure of emotional intelligence were closely related to traditional personality traits. Similarly Bar-On (1997) reported significant correlations between many sub-scales of the EQ-i and the 16 Personality Factor Questionnaire (16PF 5th edition; Cattell et al., 1999). Research by Ciarrochi et al. (2000) found that emotional intelligence, as assessed by the MEIS, correlates with empathy, but shares little overlap with extraversion and neuroticism. This would suggest that the link between emotional intelligence and personality is largely determined by the measure used. This study is partly exploratory, but three tentative hypotheses will be made.

### Self vs. observer ratings

There is an extensive literature in psychology concerned with self vs. other/observer difference in ratings of various behaviors (Conway and Huffcutt, 1997; Pronin et al., 2004; Connolly et al., 2007; Bollich et al., 2011). This is variously called multi-source feedback, 360° feedback or inter-rater reliability (Stolarova et al., 2014). A wide variety of issues have been considered such as the duration of acquaintance, observer type (peers, boss, subordinate), personality of the raters etc. and how these influence the ratings of observers and which may account for the difference between self and other. There are also a list of cognitive biases that may also account for the differences. Finally, there is also an academic literature going back well over 20 years which suggests a positive-self bias which indicates that managers tend to rate themselves overall more positively than those they work with. There appear few studies on self-other differences in emotional intelligence.

There are however various papers measured vs. self-estimated emotional intelligence which showed evidence of estimation bias (Petrides and Furnham, 2000; Siegling et al., 2014).

### Hypotheses

In line with the idea that 360 measures of EI (i.e., reports by others) are better predictors of actual performance than self report measures because of less dissimulation and other biases (poor self-insight) it is hypothesized that:

H1. 360 measures of emotional intelligence as assessed by the EQ-I show a stronger link with personality traits than self report measures of emotional intelligence.

To test the thinking that emotional competence is directly related to one's ability to manage feelings and relationships it is proposed that elements of personality measures that focus on interpersonal skills and ability to build relationships will show more overlap with emotional intelligence and as such:

H2. The OPQ “Feelings” factors will show a significant correlation with self report measures of Emotional Intelligence.
H3. The OPQ “Relationships” factors will show a significant correlation with the 360 measure of Emotional Intelligence.

## Methods

### Participants

In all 329 people took part of which 301 were males. They were all British, ranged in age from 30 to 50 years old and were senior middle managers and executives taking part on leadership development programmes working in the financial or IT sector.

### Measures

#### Bar-On emotional quotient inventory (Bar-On, 2004)

The Emotional Quotient Inventory (EQ-i), EQ-360 and EQ-i: YV were developed to assess the Bar-On model of emotional-social intelligence. The EQ-I is a self-report measure designed to measure a number of constructs related to EI. The EQ-I consists of 133 items and takes approximately 30 min to complete. It gives an overall EQ score as well as scores for the five composite scales and 15 subscales (Bar-On, 2004, 2006). In addition to the self report measure a 360 version of the tool was also administered with data collected from participants' peers. This has similar psychometric properties as the self-report table.

#### Occupational personality questionnaire

In order to assess personality, respondents completed the 230 item Occupational Personality Questionnaire (OPQ). The OPQ measures personality at three levels. First are six factors, five of which describe the “Big Five” factors plus an achievement factor. At the next level is a 16-factor solution. Third is the deductively-rather than factor analytically-derived “Concept Model” consisting of 30 scales. Subjects register their level of agreement with each statement on a five point Likert scale.

### Procedure

Participants were tested during a management training programme. They had ethical committee permission to proceed. All were told to be as honest as possible, and given feedback on their results at a later date. Furthermore, those who worked with the target person also evaluated them on the EQi. Efforts were made to get full 360 data such that the target person (who completed all the measures) was evaluated by the boss (superior), peers, reports (subordinates), and clients. Inevitably these varied greatly between individuals both in terms of the number of people who completed the observer version of the questionnaire but their specific role with respect to the target person. For the purposes of this study the observer ratings were combined to get an other/observer score. On average each person had more than two observer reports combined.

## Results

First, the data was inspected for outliers, and highly skewed distributions however, none appeared. Given the imbalance between males and females in this sample a MANOVA was run comparing the sexes on the 30 OPQ scores and the five EQi scores. Neither was statistically significant.

The total EQi score for self was 105.22 (*SD* = 16.77) while the total score for others was 100.59 (*SD* = 8.9). This was significant: [*t*_(212)_ = 3.45, *p* < 0.001].

The next step involved subjecting the 32 OPQ scales to a Promax and then Varimax rotated factor analysis. The scales failed to load onto three factors in line with the conceptual model underpinning the OPQ and therefore further analysis was carried out on all of the OPQ scale scores. Interestingly the result of this analysis indicated a five factor solution suggesting a factor model aligned with the Big 5 model of personality (Costa and McCrae, 1992). The results were similar to those of Woods and Hardy (2012) who factor analyzed the results of a similar number of British participants. The fact that the OPQ scales failed to load on the three factors does challenge the conceptual model of the test.

Next, the five factors of the EQi measure were subjected to similar factor analysis, using both orthogonal and oblique rotations. In both cases a single factor emerged with all scales loading on the single factor. Again factor analysis of this scale fails to meet theoretical expectations.

However, for comparison with other studies further analysis will be performed on the total score, and the various subscale scores.

All three hypotheses received partial support. The three OPQ facet scales (Relationships, Thinking, Feelings) were then correlated with both the self and 360 measures of the five EI scales. Tables 1–**3** below presents these correlations. Given their numbers and the possibility of Type 1 errors, Bonferroni corrections were made though tables show un-corrected correlations. Table 1 shows that the relationships element of personality correlates more significantly with the 360 measures of emotional intelligence than the self report measure although overall the correlations are low.

**Table 1:** **Pearson product-moment correlation between measures of Emotional Intelligence and OPQ Relationships Scale**.

			**Mean**	***SD***	**(SIR)**	**(SIA)**	**(SS)**	**(SA)**	**(SM)**	**(OIR)**	**(OIA)**	**(OS)**	**(OA)**	**(OM)**	**(P)**	**(CN)**	**(ON)**	**(IM)**	**(OG)**	**(A)**	**(SC)**	**(M)**	**(D)**	**(CR)**
1	Self inter	(SIR)	98.22	11.82	–																			
2	Self intra	(SIA)	103.58	17.37	0.08	–																		
3	Self stress	(SS)	105.56	11.23	−0.03	−0.00	–																	
4	Self adapt	(SA)	107.58	13.81	0.14^*^	0.09	0.06	–																
5	Self mood	(SM)	104.54	10.16	0.11	0.11	0.15^*^	0.03	–															
6	Other inter	(OIR)	97.96	10.48	0.12	0.10	0.10	0.12	0.48^**^	–														
7	Other intra	(OIA)	99.79	8.64	0.10	0.10	0.08	0.05	0.40^**^	0.24^**^	–													
8	Other stress	(OS)	102.67	6.99	0.01	0.07	0.41^**^	0.09	0.49^**^	0.45^**^	0.24^**^	–												
9	Other adapt	(OA)	102.29	7.42	0.08	0.07	0.17^*^	0.08	0.47^**^	0.50^**^	0.33^**^	0.67^**^	–											
10	Other mood	(OM)	98.55	10.53	0.11	0.12	0.15^*^	0.03	0.41^**^	0.49^**^	0.40^**^	0.49^**^	0.47^**^	–										
11	Persuasive	(P)	5.13	2.09	0.00	−0.03	0.00	−0.00	0.20	0.10	0.16	−0.09	−0.03	0.10	–									
12	Controlling	(CN)	6.42	1.69	0.05	0.10	−0.05	0.04	0.07	0.02	0.16^*^	−0.00	0.01	0.14^**^	0.35	–								
13	Outspoken	(ON)	5.79	2.13	−0.05	0.06	−0.02	0.12	0.04	−0.03	0.16^*^	−0.05	0.06	0.07	0.09	0.21	–							
14	Independent minded	(IM)	5.93	2.03	−0.19	−0.12	0.08	0.06	−0.18^**^	−0.12	−0.09	−0.13	−0.06	−0.04	−0.02	0.02	0.21	–						
15	Outgoing	(OG)	5.28	2.22	0.06	−0.01	−0.10	0.06	0.29^**^	0.26^**^	0.11	−0.06	−0.04	0.05	0.28	0.25	0.29	−0.02	–					
16	Affiliative	(A)	5.36	2.02	0.04	−0.03	−0.02	0.06	0.14^*^	0.15^*^	0.04	−0.02	−0.09	0.14^*^	0.17	0.07	0.01	−0.09	0.41	–				
17	Socially confident	(SC)	5.16	2.00	0.10	0.02	−0.04	0.05	0.29^**^	0.24^**^	0.11	0.16^*^	0.03	0.16^**^	0.29	0.17	0.03	−0.14	0.38	0.30	–			
18	Modest	(M)	5.80	4.16	−0.04	−0.02	−0.01	−0.01	−0.18^**^	−0.03	−0.10	−0.00	−0.05	−0.11	−0.22	−0.17	−0.11	0.09	−0.20	−0.09	−0.08	–		
19	Democratic	(D)	5.49	2.01	0.10	−0.09	−0.03	0.05	0.02	0.06	0.05	0.09	0.00	0.03	0.05	−0.04	−0.07	−0.29	0.03	0.17	0.10	0.03	–	
20	Caring	(CR)	5.51	1.97	0.17^**^	−0.01	−0.12	0.00	0.06	0.23^**^	−0.05	0.15^*^	0.12	0.12	−0.06	−0.07	−0.17	−0.28	−0.03	0.18	0.05	0.07	0.30	–

** p < 0.01;

* *p < 0.05*.

Table 2 shows there are more significant correlations between the OPQ Thinking scale facets and EQi than with the Relationships scale. Conventional, Rational and Forward thinking all correlate negatively with both self measures of EQi. In Table 3 the Worrying and Emotionally Controlled measures of “Feelings” correlate most significantly with both self and 360 measures of EQi although there is little other evidence to suggest a link between the OPQ Feelings scale and Emotional Intelligence. Across all 3 OPQ scales (Relationships, Thinking, Feelings) there was some evidence of a relationship with Emotional Intelligence and this was more evident with the 360 measures of EQi.

**Table 2:** **Pearson product-moment correlation between measures of Emotional Intelligence and OPQ Thinking Scale**.

			**Mean**	***SD***	**(Sir)**	**(SIA)**	**(SS)**	**(SA)**	**(SM)**	**(OIR)**	**(OIA)**	**(OS)**	**(OA)**	**(OM)**	**(R)**	**(E)**	**(B)**	**(CV)**	**(CP)**	**(I)**	**(VS)**	**(A)**	**(F)**	**(DC)**	**(CS)**	**(RF)**
1	Rational	(R)	5.22	1.90	−0.07	0.03	0.11	−0.04	−0.16	−0.19^**^	−0.07	0.01	0.01	−0.03	–											
2	Evaluative	(E)	5.59	2.45	0.01	−0.02	−0.04	−0.10	−0.07	−0.01	0.06	0.01	0.06	−0.02	0.14	–										
3	Behavioral	(B)	5.53	1.83	0.07	−0.02	−0.01	0.03	0.13	0.13	0.10	0.12	0.11	0.07	−0.09	0.04	–									
4	Conventional	(CV)	5.83	2.15	−0.09	−0.09	0.09	−0.19^**^	−0.26^**^	−0.17^*^	−0.10	−0.16^*^	−0.17	−0.07	0.16	0.03	−0.26	–								
5	Conceptual	(CP)	5.58	2.06	0.04	0.01	0.01	0.03	−0.05	0.00	−0.04	−0.05	−0.03	0.03	0.01	0.12	0.22	−0.19	–							
6	Innovative	(I)	5.52	2.08	0.08	0.03	0.00	0.08	0.09	0.01	0.13	−0.04	0.06	−0.02	−0.11	0.05	0.01	−0.36	0.31	–						
7	Variety sk	(VS)	5.78	1.92	−0.04	−0.07	0.00	0.01	0.06	−0.09	−0.02	−0.03	−0.06	0.05	−0.25	−0.02	−0.05	−0.23	0.12	0.21	–					
8	Adaptable	(A)	5.38	1.73	0.03	0.04	−0.05	−0.05	0.09	0.18^**^	−0.02	0.04	−0.03	0.07	−0.12	−0.06	0.14	−0.12	0.03	−0.11	0.02	–				
9	Forward thk	(F)	6.49	2.00	−0.00	0.01	0.03	0.07	−0.18^**^	−0.20^**^	−0.14^*^	−0.08	−0.05	−0.03	0.02	0.05	0.07	−0.09	0.16	0.18	0.03	0.15	–			
10	Detail Cons	(DC)	4.77	1.99	−0.09	0.05	0.04	−0.08	−0.14^*^	−0.04	−0.07	0.03	−0.01	−0.05	0.33	0.13	−0.20	0.37	−0.13	−0.34	−0.22	0.10	0.04	–		
11	Conscientious	(CS)	4.61	2.16	−0.01	0.04	0.08	−0.00	−0.06	0.02	0.06	0.07	0.12	−0.11	0.27	0.12	−0.11	0.30	−0.18	−0.20	−0.25	0.09	0.04	0.58	–	
12	Rule follow	(RF)	5.39	1.92	−0.06	0.07	0.06	−0.06	−0.09	−0.02	−0.06	0.00	−0.02	−0.05	0.19	0.02	−0.16	0.50	−0.08	−0.30	−0.31	0.05	0.04	0.45	0.40	–

** p < 0.01;

* *p < 0.05*.

**Table 3:** **Pearson product-moment correlation between measures of Emotional Intelligence and OPQ Feelings Scale**.

			**Mean**	***SD***	**(SIR)**	**(SIA)**	**(SS)**	**(SA)**	**(SM)**	**(OIR)**	**(OIA)**	**(OS)**	**(OA)**	**(OM)**	**RX**	**WR**	**TM**	**OP**	**TR**	**EC**	**VG**	**CV**	**A**	**DC**
1	Relaxed	(RX)	5.38	2.05	0.01	0.01	0.08	−0.02	0.10	0.08	0.01	0.17^*^	0.07	0.02	–									
2	Worrying	(WR)	5.11	2.21	−0.15^**^	−0.06	−0.08	−0.12	−0.24^**^	−0.11	−0.14^*^	−0.18^**^	−0.15^*^	−0.02	−0.33	–								
3	Tough mind	(TM)	5.21	1.88	−0.01	0.06	0.00	0.13	0.16	0.18	0.02	0.28	0.13	−10	0.27	−0.31	–							
4	Optimistic	(OP)	6.12	3.75	0.03	−0.01	0.02	−0.01	0.01	−0.10	−0.06	−0.12	−0.09	0.02	0.09	0.01	−0.04	–						
5	Trusting	(TR)	6.45	4.67	0.04	0.01	0.00	−0.03	−0.01	0.03	−0.01	−0.06	−0.02	−0.04	0.07	0.02	0.00	0.08	–					
6	Emotionally controlled	(EC)	5.63	2.06	−0.16^*^	−0.07	−0.05	−0.17^**^	−0.18^**^	−0.10	−0.22^**^	0.02	−0.06	−0.15^*^	0.03	0.28	0.04	−0.05	0.03	–				
7	Vigorous	(VG)	5.13	1.98	0.06	0.08	−0.13^*^	0.01	0.01	0.03	−0.05	−0.04	−0.08	0.01	0.00	0.09	−0.06	−0.07	−0.01		–			
8	Competitive	(CV)	5.91	2.3	−0.06	−0.13^*^	0.05	0.01	−0.04	−0.21^**^	−0.03	−0.08	−0.06	−0.07	0.03	−0.06	−0.21	0.03	−0.12	−0.08	0.08	–		
9	Achieving	(A)	5.13	2.13	0.05	0.02	−0.01	0.06	0.06	−0.04	0.04	0.03	0.02	0.03	−0.01	−0.17	0.00	−0.08	−0.09	−0.21	0.29	0.43	–	
10	Decisive	(DC)	5.98	2.02	0.07	0.03	0.04	0.08	−0.01	−0.04	0.04	−0.09	−0.02	−0.01	−0.01	−0.15	−0.13	0.05	−0.02	−0.15	0.07	0.07	0.04	–

** p < 0.01;

* *p < 0.05*.

Table 4 shows the self and other scores as well as *t*-test results for each analysis. Three things are noticeable from these data. *First*, nearly all (16 out of 21) analyses showed a significant difference all in the same direction. Overall participants thought that they were more emotionally intelligent than did observers. The only exception was the interpersonal total scale and sub-scale scores where the lack of significant difference seems mainly attributable to the participants giving slightly below average scores. *Second*, participants tended to give themselves scores between a third and a half of a standard deviation above the mean while observers gave scores one of two points above or below the mean of 100. *Third*, in nearly all instances the SD of the observers were lower than that of the estimates of the participants.

**Table 4:** **Self and other rated EQi scores**.

	**Self**		**Other**		**Difference**	***t***
	***X***	***SD***	***X***	***SD***		
**Intrapersonal total**	103.58	17.37	99.79	8.64	5.80	4.79^***^
IA1. Self-regard	106.43	9.20	100.63	7.83	5.79	9.14^***^
IA2. Self-awareness	102.18	11.91	96.92	8.74	5.26	4.29^***^
IA3. Assertiveness	106.39	12.78	101.92	8.73	4.47	5.36^***^
IA4. Independence	105.77	12.73	99.81	8.76	5.96	6.83^***^
IA5. Self-actual	104.39	10.51	98.62	8.68	5.77	6.67^**^
**Interpersonal total**	98.22	11.82	97.96	10.48	0.26	0.29
IE1. Empathy	98.46	12.71	97.91	8.48	0.56	0.67
IE2. Social responsibility	99.45	11.91	97.99	11.73	1.48	1.69
IE3. Interpersonal relations	97.65	12.53	98.79	9.12	−1.13	1.49
**Stress management total**	105.56	11.23	102.67	6.99	3.29	4.58^**^
S1. Stress tolerance	108.55	10.38	102.67	6.99	5.58	7.69^***^
S2. Impulse control	101.80	12.41	101.53	7.91	0.27	0.39
**Adaptability**	107.58	13.81	102.29	7.42	5.29	7.36^***^
A1. Reality testing	105.67	10.87	102.87	10.87	2.82	3.90^***^
A2. Flexibility	107.80	11.64	101.32	11.42	6.48	8.07^***^
A3. Problem solving	105.32	10.35	101.68	6.73	3.03	5.19^**^
**General mood**	104.54	10.16	98.55	10.53	5.57	7.98^***^
GM1. Optimism	104.84	9.90	99.40	7.53	5.44	7.91^***^
GM2. Happiness	103.89	10.84	98.92	8.80	4.96	7.03^***^
**Total**	104.54	17.23	100.48	7.74	4.06	3.45^***^

*** p < 0.001;

** *p < 0.01*.

Significant correlations on each of the three OPQ scales were regressed against EQi. Six, step-wise multiple regressions were computed with the Self 360 EI score and the five subscales as the criterion variable and the 9 significant OPQ scales as the predictor variables.

Following this a series of regression were performed with the total and five EI scales as the criterion variable and all nine OPQ scores as the predictor variables. The aim was to examine which OPQ facets were most strongly linked to the EI facets.

Four regressions were significant: the Independent Minded scale on the OPQ was a significant predictor of the total self-report EI score and accounted for 4% of the variance. The regression which accounted for most of the variance (17%) was the Mood factor and it showed that higher scores on Independent Minded and Outgoing and low scores on Conventional were associated with high scores on Mood Intelligence as. Higher scores on Outgoing were associated with higher scores on Mood Intelligence. The Stress subscale did not show any significant relationships with personality. On the Intrapersonal subscale, there was a significant negative relationship with Independent Minded but this accounted for a very small amount of variance (less than 1%).

A series of regressions were then computed similar to those reported in Table 5 however this time the criteria variable was the observer rather than the self-reported scores on the EQ1. These are shown in Table 6.

**Table 5:** **Results of the Regression with EI self total and subscale scores as the criterion (independent) variables and the significant OPQ (dependent) variables as the predictor**.

		**Total**		**Intrapersonal**		**Interpersonal**		**Stress manag**		**Adapt**		**Mood**	
		**Beta**	***t***	**Beta**	***t***	**Beta**	***t***	**Beta**	***t***	**Beta**	***t***	**Beta**	***t***
1	Independent minded	−0.13	2.01^*^	−0.14	2.06^*^	−0.158	2.40^*^	0.07	1.03	0.05	0.70	−1.77	2.66^**^
2	Outgoing	0.04	0.50	−0.04	0.54	0.00	0.02	−0.11	1.46	−0.01	0.13	0.19	2.65^**^
3	Socially confident	0.04	0.56	−0.03	0.36	0.02	0.23	−0.02	0.22	−0.02	0.31	0.11	1.46
4	Caring	0.06	0.84	−0.02	0.30	0.13	1.90	−0.11	1.61	0.02	0.26	0.01	0.14
5	Behavioral	−0.06	0.82	−0.06	0.87	−0.02	0.32	0.02	0.23	−0.06	0.95	0.02	0.32
6	Conventional	−0.11	1.61	−0.12	1.70	−0.07	1.07	0.13	1.82	−0.17	2.45	−0.19	−2.70^**^
7	Tough minded	0.00	0.01	0.05	0.72	−0.05	0.78	−0.02	0.24	0.13	2.01	0.09	1.39
8	Worrying	−0.14	1.82	−0.01	0.14	−0.11	1.41	−0.11	1.50	−0.00	0.03	−0.08	1.11
9	Emotionally controlled	−0.03	0.45	−0.07	1.00	−0.12	1.54	−0.08	1.20	−0.17	2.50	−0.04	−0.50
*F*_(9, 238)_		2.20^*^		0.99		2.61^**^		1.54		2.31^*^		6.10^***^	
Adjusted R Square		0.04		0.00		0.06		0.02		0.05		0.17	

*** p < 0.001;

** p < 0.01;

* *p < 0.05*.

**Table 6:** **Results of the Regression with EI 360 total and subscale scores as the criterion (independent) variables and the significant OPQ (dependent) variables as the predictor**.

		**Total**		**Intrapersonal**		**Interpersonal**		**Stress manag**		**Adapt**		**Mood**	
		**Beta**	***t***	**Beta**	***t***	**Beta**	***t***	**Beta**	***t***	**Beta**	***t***	**Beta**	***t***
1	Independent minded	−0.13	1.89	−0.11	1.51	−0.05	0.76	−0.09	1.31	−0.06	0.76	−0.18	2.66^**^
2	Outgoing	0.10	1.41	0.02	0.22	0.22	2.93^**^	−0.12	1.62	−0.06	0.81	0.19	2.65^**^
3	Socially confident	0.10	1.39	0.04	0.54	0.09	1.14	0.12	1.53	−0.04	0.51	0.12	1.46
4	Caring	0.10	1.48	−0.11	1.46	0.22	3.23^***^	0.12	1.67	0.10	1.35	0.01	0.14
5	Behavioral	0.07	0.10	0.06	0.80	0.03	0.40	0.03	0.45	0.03	0.43	0.02	0.32
6	Conventional	−0.19	2.76^**^	−0.05	0.67	−0.10	1.47	−0.15	2.10^*^	−0.15	1.98	−0.19	2.70^**^
7	Tough minded	0.09	1.34	−0.00	0.01	0.15	2.20^*^	0.22	3.10^**^	0.09	1.28	0.09	1.40
8	Worrying	−0.06	0.78	−0.02	0.18	−0.01	0.17	−0.04	0.56	−0.08	0.96	−0.08	1.11
9	Emotionally controlled	−0.11	1.60	−0.18	2.40	0.02	0.21	0.05	0.63	−0.03	−0.37	−0.04	0.50
*F*_(9, 202)_		5.81^***^		1.62		5.01^***^		4.04^***^		1.71		6.11^***^	
Adjusted R Square		0.17		0.03		0.15		0.11		0.03		0.17	

*** p < 0.001;

** p < 0.01;

* *p < 0.05*.

Four regressions were significant: the conventional scale on the OPQ was a significant predictor of the total 360 EI score and accounted for 17% of the variance. Similarly with the Interpersonal factor, Outgoing, Tough minded and Caring accounted for 15% of the variance and for the Mood factor, Independent Minded, Outgoing and Conventional accounted for 17% of the variance. The regression which accounted for most of the variance (17%) was the Mood factor and it showed that higher scores on Independent Minded and Conventional were associated with lower scores on Mood Intelligence as assessed. Higher scores on Outgoing were associated with higher scores on Mood Intelligence. Two factors predicted the Stress subscale; Tough Minded was associated with a higher ability to Manage Stress and Conventional was associated with a lower ability to manage stress. The intrapersonal scale did not report any significant relationships with personality.

## Discussion

Factor analyses of both scales failed to confirm theoretical expectations. This could be a function of this particular modest-sized and relatively homogeneous sample. Yet previous studies have shown the same thing: namely failure to confirm the theoretical structure of the two models and psychometric tests used in this study. Next, the data do suggest modest overlap between these two measures but clearly more some scales more than others. Few, if any correlations exceeded *r* = 0.30 and fewer than half were significant.

One of the most interesting issues was self-other differences in the EQ scores. Table 4 shows that of the 21 self-other differences participants rated themselves significantly higher on all but five. The biggest differences were on Flexibility, Independence and Self-regard. Participants gave themselves highest rating for stress tolerance. There are numerous possible explanations for these findings. It is possible that the participants gave inflated scores either because they were dissimulating to try to create a good impression in a work setting or else because they lacked self-awareness into their actual abilities and behavior. Equally it is possible but unlikely that observer ratings were lower either because they did not feel the pressure to do impression management or else that they did not have sufficient data on the person they rated. Indeed, there is a large literature on self-serving bias which suggests that compared to both objective and observer data, people tend to over-estimate their abilities (Furnham, 2008a).

There are two further interesting features of this part of the study. The first is the exception of the interpersonal EQi total and subscale scores where there was no difference between self- and other-reports possibly because of the fact that the participants scored themselves below average on this scale. This component and subscale scores indicate Social Adeptness, the Ability to Understand Others, and to interact and relate well to people. It is interesting, but unclear, why this sample scored themselves consistently lower on this component and scales, particularly on Interpersonal Relations. The second is the size of the difference which is, on average, around a third of a standard deviation.

The 360 scores on Emotional Intelligence also reported a stronger link with personality than the self report measure. In Table 5 only 4% of the variance in Total Self scores on Emotional Intelligence was accounted for by personality vs. Seventeen percent of the variance in Total Other scores. This suggests that measures of emotional intelligence which include a 360 component may provide a more useful indicator of an individual's ability to manage own feelings and those of others.

The second aspect of the study was exploring the link between personality and Emotional Intelligence. The Relationship scale on the OPQ produced a greater number of significant relationships than the Feelings and Thinking scale. This indicates that those individuals with a natural ability to manage relationships are perceived by others to be more emotionally intelligent. In particular it would appear that those who are Socially Confident and exhibit a caring nature are regarded more highly in terms of emotional intelligence by their colleagues. On the other hand there was little relationship between an individual's score on the “Feelings” component of the OPQ and emotional intelligence suggesting that those elements of personality related to the management of feelings may not be linked to ability measures of emotional intelligence such as the Bar-on. This would support the argument that emotional intelligence offers little more than a measure of personality: that is, the incremental benefits of using *this* EQ measure over *this* personality test is poor.

The component of emotional intelligence which showed the greatest linked with personality on both self and 360 data was the Mood Scale accounting for 17% of the variance in both instances. The link between mood and personality has been studied extensively with most studies assuming that personality influences mood (Harris and Lucia, 2003; Brown et al., 2011). Some researchers including Zajenkowski et al. (2012) challenge this view and suggest that the correlations could in fact be interpreted in the opposite direction whereby self-report personality measures are influenced by an individual's mood in particular situations. In the current study though the fact that both the 360 and self-report measure correlated highly with personality would give weight to the more common view that mood is significantly affected by personality.

Like all studies this one has limitations. We were unable to obtain data on the participant's age and job experience which may have been relevant to their individual scores. More importantly we were unable to examine the internal reliability of the 360 “other” reports which may mean that that data is potentially unstable. Furthermore, despite that both measures are used extensively in business settings (Furnham, 2008b) it would be most desirable to relate both tests to objective work outcome measures attempting to establish which test and which scales have strongest predictive validity, and indeed the incremental validity of the one test over the other. Another limitation of this study is the external validity, i.e., whether the conclusion could be generalized to the general population since all the sample subjects came from a business setting.

### Conflict of interest statement

The authors declare that the research was conducted in the absence of any commercial or financial relationships that could be construed as a potential conflict of interest.
